# A Training Proposal to Improve Multidirectional Running Technique in Male and Female Handball Players: A Pilot Study

**DOI:** 10.3390/ijerph18042056

**Published:** 2021-02-20

**Authors:** Carmen Ferragut, Román Pedreira, José Julio Espina, Helena Vila

**Affiliations:** 1 Faculty of Medicine and Health Science, University of Alcalá, Research Group GRIGEDE, 28871 Alcalá de Henares, Spain; cferragutfiol@gmail.com; 2 Faculty of Education, University of Vigo, 36905 Pontevedra, Spain; romanpedreira19@hotmail.com; 3 Faculty of Education, University of Alicante, 03690 San Vicente del Raspeig, Spain; jj.espina@ua.es; 4 Faculty of Education, University of Vigo, Research Group HealthyFit, 36905 Pontevedra, Spain

**Keywords:** team sports, agility, change of direction, power

## Abstract

Multidirectional running has been described as an important factor in team sports performance. The aim of the present study was to determine changes in T-test, 505 time, 10 m sprint, squat jump (SJ), countermovement jump (CMJ), countermovement jump right leg (CMJRL), and countermovement jump left leg (CMJLL) following exposure to 12 sessions over 4 weeks of a multidirectional running sprint training intervention in male and female handball players. A total of 31 handball players (15 male and 16 female) were recruited for this study and then randomly assigned to an experimental group (EG) or control group (CG). Male EG players showed improvements in 505 Preferred Side (PS) (*p* ≤ 0.05), 505 Non-Preferred Side (NPS) (*p* ≤ 0.05), and 10 m sprint (*p* ≤ 0.05), while female EG players presented statistically significant improvements between pre- and post-test for the T-test (*p* ≤ 0.05), 505 PS (*p* ≤ 0.05), 505 NPS (*p* ≤ 0.05), and 10 m sprint (*p* ≤ 0.05). No statistically significant pre- and post-test differences were observed in CG (all *p* ≥ 0.05) or between male and female players. We found an improvement in handball players’ agility and speed of movement following the intervention protocol, suggesting the need to introduce this program into our training sessions. It may also be necessary to select and develop more specific tests in order to evaluate multidirectional work in handball players.

## 1. Introduction

In many team sports such as handball, rapid, decisive changes of direction or speed can result in a break or a score during the match and can therefore be considered a critical factor for successful performance [1,2,3]. As a result, change of direction (COD) ability has been widely investigated in team sports [1,4,5].

COD can be defined as the ability to quickly change direction while sprinting. Several authors have reported that COD is the physical foundation of agility, as it incorporates the mechanism associated with agility performance (i.e., deceleration, directional change, and acceleration) [6]. Thus, Sheppard and Young [6] have described several factors that can be considered relevant in determining COD ability, including technical speed and leg muscle qualities.

Regarding this latter factor, several studies have attempted to improve COD performance through resistance training, implementing protocols such as heavy lifts [7], Olympic-style lifts [8], and plyometrics [9,10], and through sprint training [3,11]. However, the findings have been inconsistent, with some studies reporting a poor or non-significant relationship between COD speed and linear speed or muscle strength. Recently, Chabeene et al. (2018) [12] found that strength training with accentuated eccentric muscle actions showed small to large size effects on COD speed performance in athletes, but further research is required.

As mentioned earlier, technique has been cited as an important component of COD ability, but surprisingly, there is little empirical evidence to support this assertion [2,13]. Most of the research on this question has been conducted from the perspective of injury [14]. Wheeler and Sayers [2] used an agility test to study rugby players, examining differences in agility (side-stepping maneuvers) and running technique between reactive and pre-planned performance conditions during a rugby-specific task. They focused on foot placement patterns during the test, seeking to determine the difference between planned and pre-planned conditions, and found that the presence of decision-making changed foot placement patterns from pre-planned conditions. Unfortunately, no studies provide coaches with empirical knowledge on how to optimize COD technique.

It is also important to note that what makes an athlete quick on the court is often the ability to change direction faster than the opponent; similarly, athletes who possess the ability to decelerate and accelerate again quickly usually perform the fastest in multidirectional speed [15,16]. Given the above, it appears to be necessary to design training routines that genuinely improve multidirectional speed in order to obtain a better performance from athletes during the match.

The aim of the present study was to analyze the effects of 12 sessions of multidirectional running speed training in male and female handball players.

## 2. Materials and Methods

### 2.1. Experimental Approach to the Problem

This study used a repeated-measures design to determine changes in T-test, 505 time, 10 m sprint, squat jump (SJ), countermovement jump (CMJ), countermovement jump right leg (CMJRL), and countermovement jump left leg (CMJLL) following exposure to 12 sessions over 4 weeks of a multidirectional running sprint training intervention in male and female handball players. Subjects were divided into 4 groups: male experimental group (MEG), female experimental group (FEG), male control group (MCG), and female control group (FCG). All groups were tested before and after the 12 training intervention sessions.

### 2.2. Subjects

A total of 31 handball players (15 male and 16 female) were recruited for the study. Male players were playing in the 1^st^ National Spanish Division while female players were playing in the Galician Territorial Division. All players were randomly assigned to an experimental group (EG) or control group (CG). Table 1 gives the physical characteristics and distribution of the sample.

### 2.3. Ethics

All players aged over 18 years old, or the legal guardians for those aged under 18 years old, were notified of the potential risk involved and gave their written informed consent. This study was approved by the Vigo University Committee (code: 04–719) on research involving human subjects and was conducted in accordance with the Declaration of Helsinki.

### 2.4. Procedures

*Testing Protocol.* All tests were performed indoors under the same ambient air conditions. Athletes were familiarized with the test procedures in the week before pre-testing to minimize the learning effect between pre-test and post-test. We held two testing sessions in this study. On the first day, we collected anthropometric data and tested the countermovement jump (CMJ) and squat jump (SJ). Forty-eight hours later, participants performed the multidirectional running test. After anthropometric assessment, subjects commenced with a standardized 15-min warm-up procedure consisting of submaximal running plus change of direction exercises, multi-jump exercises, and sprints.

*Anthropometric assessment.* Height and weight measurements were taken on a level platform scale (Seca, Barcelona, Spain) with an accuracy of 0.001 m and 0.01 kg, respectively.

*Jumping performance.* To evaluate players’ dynamic leg power, the SJ and unilateral and bilateral CMJ were measured using a contact mat (Chronojump Bosco test, Barcelona, Spain)[17]. Athletes performed 2 trials separated by a 2-min recovery period. The best attempt was used for subsequent statistical analysis. Athletes were instructed to keep their hands on their hips and to land with their legs straight.

*Assessment of linear speed*. Linear speed performance was assessed as previously described by Nimphius et al. [4], but only for 10 m. Participants performed 3 trials with a 3-min rest interval between attempts. The mean time of the 3 trials was used for subsequent analysis. If the subject rocked backward or forward prior to starting, the trial was discarded and repeated after a rest interval.

*T-test.* We used the method described by [18]. Subjects began with both feet behind starting point A. At their own discretion, each subject sprinted forward 9.14 m (10 yd) to point B and rang a bell at the base of a cone with the right hand. They then shuffled 4.57 m (5 yd) to the left and touched a bell at the base of a cone (C) with the left hand before shuffling 9.14 m to the right and touching a bell at the base of a cone (D) with the right hand. Next, they shuffled 4.57 m to the left, back to point B, and touched a bell with the left hand. Subjects then ran backward, passing the finishing line at point A. Subjects were instructed not to cross their feet when side-shuffling, and if they did so, the trial was stopped and reattempted after the requisite rest period. Time was recorded from the moment subjects crossed the start/finish line at the beginning until they crossed it again with the final back pedal step. Three test trials were performed, with three minutes of recovery between trials.

*Assessment of change of direction speed.* Participants performed a 505 test following [4] with minor modifications. Subjects began from a standing start, as in the linear speed test, with their feet 30 cm behind the starting line. They were instructed to sprint to a line marked 5 m from the start line (line B) and continue sprinting toward a line placed 5 m away from line B and 10 m away from the starting line (line A), placing either their left foot (LF) or right foot (RF) on the line depending on the trial, before turning 180º and sprinting back 5 m to the finish. Time started when the player crossed line B and stopped when the player returned to line B.

If the subject changed direction during the test before hitting the turning line, or turned off the incorrect foot, the trial was disregarded and the subject completed another trial after a rest period. Two trials were recorded for turns off the left and right foot, with three minutes of recovery between trials. The best time of the two trials was used for subsequent analysis.

*Training intervention.* The training intervention (PT) was conducted in February and March 2019. The EG participated in a 4-week program comprising 3 sessions per week. These sessions were integrated into the EG’s regular handball training routine after warm-up. In order to avoid the effect of fatigue, at least 48 h elapsed between each PT session. Each handball session lasted between 70 and 90 min while the integrated PT session lasted 10 min. The PT was designed to enhance multidirectional displacement. To this end, multidirectional displacement was divided into three skills: acceleration, backward acceleration, and change of direction. The exercises selected in order to enhance these skills were as follows: ankle mobility, first step acceleration, shuffle, crossover, back pedal, and COD, based on the different skills proposed by experts. Table 2 shows the training program.

### 2.5. Statistical Analyses

We calculated the descriptive statistics for each outcome variable, including means and standard deviations, and conducted tests for normality and homogeneity. The percentage of change from pre- to post-training was calculated for all variables.

To detect differences in measures between pre- and post-test in male and female players, we used the paired T-test to test for a difference in central location (mean) between the paired samples (within group). To test for a difference in central location (mean) between groups, the independent sample T-test was applied. Differences were considered significant at *p* ≤ 0.05, and the results are expressed as mean and SD. The 95% confidence interval (95% CI) was also calculated for all measures.

Effect sizes were calculated using Cohen’s d (small effect size; d = 0.2–0.3, medium effect size; d = 0.5, large effect size, d = 0.8). Power is also reported where appropriate.

(1) $$r.:\;d/\sqrt{d^{2}+4}$$

Statistical analyses were performed in IBM-SPSS v.25 (IBM Co., Armonk, NY, USA) and an alpha level of 0.05 was set as the criterion for statistical significance in all analyses.

## 3. Results

There were no statistically significant baseline differences between EG and CG in either male or female players (all *p* ≥ 0.05).

**Figure 1:**
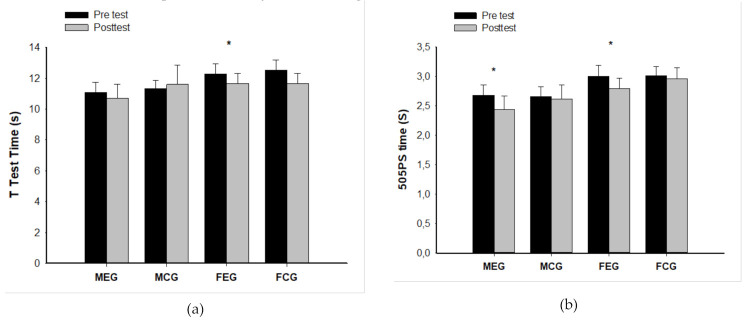

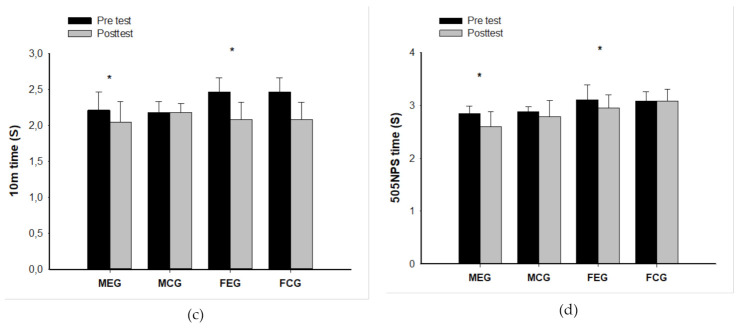
Differences between pre and post-test in (**a**) T-test, (**b**) 505 Preferred Side (PS), (**c**) 10 m time, and (**d**) 505 Non-Preferred Side (NPS) in male and female players. Legend: MEG: Male experimental group; MCG: Male control group; FEG: Female experimental group; FCG: Female control group. * *p* ≤ 0.05. Regarding the % of change in each variable, no differences were found between male and female players (all *p* ≥ 0.05).

A within-groups analysis for EG males revealed improvements in 505 Preferred Side (505 PS) (2.68 ± 0.17 vs 11.60 ± 1.26; *p* ≤ 0.05; d = 1.62). Male players also presented significant differences in 505 Non-Preferred Side (505 NPS) 2.84 ± 0.15 vs 2.60 ± 0.28 s; *p* ≤ 0.05; d = 1.35) and 10 m sprint (2.21 ± 0.25 vs 2.04 ± 0.29 s; *p* ≤ 0.05; d = 0.91). No statistically significant differences were found in CG between pre and post-test (all *p* ≥ 0.05). It is interesting to note that these significant differences were accompanied by large effect sizes (Figure 1).

Regarding female players, a within-groups analysis for EG showed significant differences between pre- and post-test in the T-test (12.29 ± 0.65 vs 11.66 ± 0.64 s; *p* ≤ 0.05; d = 1.38), and 505 PS (3.00 ± 0.18 vs 2.79 ± 0.18 s; *p* ≤ 0.05; d = 1.61), with an improvement in 505 NPS (3.11 ± 0.28 vs 2.95 ± 0.25 s; *p* ≤ 0.05; d = 0.87), and 10 m sprint (2.46 ± 0.20 vs 2.08 ± 0.24 s; *p* ≤ 0.05; d = 2.22). It is interesting to note that these significant differences were accompanied by large effect sizes. No differences were found in CG between pre- and post-test for any variables (Figure 1).

## 4. Discussion

The aim of this study was to analyze the effect of 12 sessions of multidirectional running speed training in male and female handball players. The main goal was to determine whether our players (male and female) improved in 505 and 10 m tests following a multidirectional running training intervention.

With respect to the T-test, we found no differences between pre- and post-test in male players. There are several possible explanations for this. First, the T-test may be too long and thus may not present sufficient reliability/specificity for handball players in particular and court sports in general, because the total sprinting distance covered is approximately 40 m whereas it is well documented that sprint duration in handball is between 2 and 3 seconds. Thus, improvements might have been greater if shorter distances had been used for the test [19]. Our results may also have been influenced by the need to bend down and touch the cone or by use of the back pedal drill, since neither of these movements featured extensively in our training program. Similarly, the duration of the training program may have been insufficient.

However, we did observe differences in the T-test in female players, perhaps due to their lower playing level. It has been reported that the highest level players accelerate and change direction faster than their lower-level counterparts [20]. This suggests to us that if we had increased the number of sessions and the number of “bend down” and “back pedal” drills, we might have found greater differences in female players and differences in male handball players.

Our players obtained lower scores in the T-test than those reported by Pereira [21], but their scores were similar to those published by Chaabene [22]. Given that the T-test can discriminate between low and high levels of sport performance [18], one explanation for these differences may be that Pereira’s sample consisted of male elite players whereas Chaabene’s sample consisted of young female players and was therefore more similar to our own female players.

The 505 test scores improved after training in both the male and female groups. These results may be due to improvements in first step acceleration, crossover, and most especially COD drills, all of which featured extensively in our training program. However, changes in COD ability have been associated with improvements in kinetic and COD technique factors [13,23], whereas our training program focused on kinematic factors such as step length and frequency. It is possible that we might have found larger differences if we had implemented training for longer. Thus, training duration (only 4 weeks) may have negatively affected the potential benefits of the training program.

In relation to our male players’ scores for 505 PS and 505 NPS, these presented similar values to those reported by Nimphius et al. [4] in experienced cricket players, but were lower than those reported by [13] in elite soccer players. These differences may have arisen because the 505 tests performed by Beato’s subjects were longer (15 m vs 10 m) than our 505 tests.

With regard to our female players’ scores in 505 tests, these presented similar values to those of slower basketball players in Spiteri’s study, but were worse than those of faster basketball players in the same study. This may be because our sample consisted of young female players whereas Spiteri’s sample consisted of elite female basketball players; thus, our female players obtained scores similar to the slower players in Spiteri’s study.

Both our male and female players obtained higher scores for 10 m in post-test than in pre-test. These results may be due to first step acceleration drills, which featured extensively in our training program. Our players’ results are not in line with those published by [5,13] and are more similar to those reported by [22] in young female players, albeit our female players obtained slightly worse results than Chaabene’s due to their lower playing level.

No pre- and post-test differences were detected for SJ or unilateral and bilateral CMJ in either sex. It may be that the CMJ and SJ were not the most appropriate tests for our training program. Although COD ability is significantly related to neuromuscular performance [21], two things should be borne in mind: first, in our training program we did not work on lower limb explosive power but on multidirectional displacement, and second, lower limb strength may explain only a small percentage of sprints with change of direction [24].

No differences were found between sexes in percentage of improvement, indicating that multidirectional displacement may be a good training strategy for both sexes.

## 5. Conclusions

In conclusion, we observed an improvement in agility and speed of movement in handball players following the intervention protocol proposed in this study. This finding indicates the need to introduce this program into our training sessions. It may be necessary to select and develop more specific tests in order to evaluate multidirectional work in handball players. The results of this study demonstrate that a multidirectional training routine based on specific drills effectively improves measures of multidirectional speed. Because multidirectional changes play a key role in handball, coaches and researchers should focus more on developing and implementing innovative and specific multidirectional displacement training strategies better adapted to handball. Researchers should also seek to identify more specific tests to assess multidirectional displacement in handball.

## Figures and Tables

**Table 1:** Player characteristics by groups.

	MEG (*n* = 8)	MCG (*n* = 7)	FEG (*n* = 9)	FCG (*n* = 7)
Age (yrs)	24.25 ± 6.94	24.24 ± 4.98	17.89 ± 2.93	19.71 ± 3.54
Height (cm)	183.84 ± 6.55	177.81 ± 7.37	164.18 ± 6.51	166.13 ± 4.36
Body mass (kg)	80.21 ± 9.00	84.66 ± 22.53	58.71 ± 7.03	68.76 ± 12.25
Experience (yrs)	15.75 ± 6.13	16.57 ± 5.22	8.44 ± 1.50	7.43 ± 2.15

MEG: Male experimental group; MCG: Male control group; FEG: Female experimental group; FCG: Female control group.

**Table 2:** Characteristics of the multidirectional running training.

Session	Task	Exercises × Sets × Reps	Rest Intervals	Session	Task	Exercises × Reps × Sets	Rest Intervals
Session 1	FSA	AM × 5 rep 1 × 2 FSA(1) × 10 rep t. × 2 FSA(2) × 2 rep 1 × 3 FSA(3) × 4 rep 1 × 2 FSA(4) × 4 rep 1 × 2 FSA(6) × 3 rep 1 × 1	0(s)–0(s) 0(s)*–*20(s) 0(s)*–*20(s) 0(s)*–*20(s) 0(s)*–*20(s) 30(s)	Session 2	SF	AM × 5 rep 1 × 2 SF(1) × 5 rep 1. × 2 SF(2) × 4 rep 1 × 2 SF(3) × 3 rep 1 × 2 SF(6) × 4 rep 1 × 2 SF(4) × 1 rep 1 × 2	0(s)*–*0(s) 0(s)*–*20(s) 3(s)*–*20(s) 5(s)*–*20(s) 0(s)*–*20(s) 10(s)*–*20(s)
Session 3	CO SF	AM × 5 rep 1 × 2 CO × 3 rep 1 × 2 CO(1) × 2 rep 1 × 2 CO × 3 rep 1 × 2 SF+CO × 2 rep 1 2SF+CO × 2 rep 1	0(s)*–*0(s) 10(s)*–*20(s) 10(s)*–*20(s) 10(s)*–*20(s) 30(s)30(s)	Session 4	FSA	AM × 5 rep 1 × 2 FSA(2) × 2 rep 1. × 3 FSA(3) × 4 rep 1 × 1 FSA(4) × 4 rep 1 × 1 FSA(5) × 3 rep t × 1 FSA(6) × 3 rep t × 1	0(s)*–*0(s) 0(s)*–*20(s) 3(s)*–*20(s) 3(s)*–*20(s) 30(s) 30(s)
Session 5	SF	AM × 5 rep 1 × 2 SF(3) × 3 rep t. × 2 SF(6) × 3 rep 1 × 2 SF(7) × 6 rep 1 × 2 SF(4) × 1 rep 1 × 2	0(s)*–*0(s) 5(s)*–*20(s) 0(s)*–*20(s) 0(s)*–*20(s) 10(s)*–*30(s)	Session 6	CO SF	AM × 5 rep 1 × 2 CO × 3 rep 1 × 2 CO(1) × 2 rep 1 × 2 CO × 3 rep 1 × 2 SF+CO × 2 rep 1 2SF+CO × 2 rep 1	0(s)*–*0(s) 10(s)*–*20(s) 10(s)*–*20(s) 10(s)*–*20(s) 30(s) 30(s)
Session 7	FSA	AM × 5 rep 1 × 2 FSA(3) × 4 rep 1. × 1 FSA(4) × 4 rep 1 × 1 FSA(7) × 3 rep t × 1 FSA(6) × 3 rep t × 1	0(s)*–*0(s) 3(s)*–*20(s) 3(s)*–*20(s) 30(s) 30(s))	Session 8	CO SF	AM × 5 rep 1 × 2 CO × 3 rep 1 × 2 SF+CO × 2 rep 1 2SF+CO × 2 rep 1 SF(5)+CO × 2 rep 1	0(s)*–*0(s) 10(s)*–*20(s) 30(s) 30(s) 30(s)
Session 9	BP COD	AM × 5 rep 1 × 2 BP(1) × 5 rep t BP × 5 rep t COD (1) × 3 rep 1 COD × 3 rep 1	0(s)*–*0(s) 30(s) 30(s) 30(s) 30(s)	Session 10	FSA	AM × 5 rep 1 × 2 FSA(3) × 4 rep 1. × 1 FSA(4) × 4 rep 1 × 1 FSA(8) × 3 rep t × 1 FSA(6) × 3 rep t × 1	0(s)*–*0(s) 3(s)*–*20(s) 3(s)*–*20(s) 30(s) 30(s)
Session 11	SF CO FSA	AM × 5 rep 1 × 2 2SF+CO × 1 rep 1 CO+FSA × 1 rep 1 SF+CO+FSA × 1 rep 1	0(s)*–*0(s) 30(s) 30(s) 30(s)	Session 12	BP COD	AM × 5 rep 1 × 2 BP(1) × 5 rep t BP × 5 rep t COD (1) × 3 rep 1 COD × 3 rep 1	0(s)*–*0(s) 30(s) 30(s) 30(s) 30(s)

Legend: AM: Ankle Mobility, FSA = First Step Acceleration; CO = Crossover; SF = Shuffle; BP = Back Pedal; COD = Change of Direction; rep l. = repetitions each side; rep t. = Total repetitions.

## Data Availability

The data presented in this study are available on request from the corresponding author. The data are not publicly available due to privacy.
